# The Effect of an Autoimmune Protocol (AIP) Diet in Adults With Rheumatoid Arthritis: A Single Arm Crossover Pilot Feasibility Study

**DOI:** 10.1002/msc.70214

**Published:** 2026-04-04

**Authors:** Julianne J. McNeill, Gael J. Mearns, Rebecca Grainger, Caryn Zinn

**Affiliations:** ^1^ School of Sport & Recreation, Human Potential Centre, Faculty of Health and Environmental Sciences Auckland University of Technology Auckland New Zealand; ^2^ School of Clinical Sciences, Faculty of Health and Environmental Sciences Auckland University of Technology Auckland New Zealand; ^3^ Department of Medicine University of Otago Wellington Wellington New Zealand

**Keywords:** AIP diet, autoimmune protocol diet, nutrition, patient‐reported outcomes, RAID, RAPID3, rheumatoid arthritis

## Abstract

**Background:**

Individuals with rheumatoid arthritis (RA) often ask whether dietary changes or food exclusions can reduce symptoms. The Autoimmune Protocol (AIP) diet, an elimination‐based approach, is popular on social media, with anecdotal reports of benefit, but lacks clinical evaluation in RA.

**Objectives:**

To assess the feasibility and effectiveness of an AIP diet on patient‐reported outcomes (PROs) in adults with RA.

**Methods:**

In this single‐arm, open‐label pilot study, nine adults with RA followed their usual diet for 4 weeks and then an 8‐week AIP diet in a free‐living context. Weekly PRO measures included the Routine Assessment of Patient Index Data 3 (RAPID3) and RA Impact of Disease (RAID) as primary outcomes. Secondary outcomes included 3‐day weighed food records and biometric measurements (weight and waist circumference) collected at baseline and every 4 weeks. Adherence and ease of implementation were recorded weekly.

**Results:**

Nine participants (7 females, 2 males, aged 40–63 years) completed the intervention. From baseline to end week 12, mean RAPID3 scores decreased from 2.73/10 (range 0.2–5.37) to 0.99 (0–2.5). Four participants demonstrated a clinically meaningful reduction and three others reached remission from low disease activity. Mean RAID scores decreased from 3.13/10 (0.1–6.35) to 1.02 (0–2.96). Fatigue, sleep and pain improved. Mean body mass index reduced from 26.3 kg/m^2^ to 24.5 kg/m^2^. The diet was feasible with high adherence.

**Conclusions:**

An 8‐week AIP diet was associated with improvements in PROs in seven of nine participants. These pilot findings support further controlled trials to confirm efficacy and explore mechanisms as a potential adjunct therapy for RA.

**Trial Registration:**

Number ACTRN12624001391561. https://www.anzctr.org.au/ACTRN12624001391561.aspx

## 1 Introduction

Rheumatoid arthritis (RA) is a chronic systemic autoimmune disease affecting an estimated 17.6 million people globally, with a disproportionate burden on women and increasing prevalence with age (Vos et al. 2020). The goal of RA treatment is to effectively control immune‐driven inflammation to prevent progressive joint damage, with a goal of therapeutic remission (Smolen 2016). Despite advances in both biologic and non‐biologic disease‐modifying anti‐rheumatic drugs (DMARDs), remission rates in clinical studies vary widely, ranging from 5% to 43% (Ajeganova et al. 2013; Ostor et al. 2022). Furthermore, over 50% of people with RA continue to report poor or very poor health, which correlates with higher disease activity (Tański et al. 2022). Persistent fatigue, suboptimal mental health, sleep disturbances, and unacceptable levels of pain remain common unmet needs, highlighting the importance of alternative approaches for symptom management (Tański et al. 2022; Taylor et al. 2016; van Steenbergen et al. 2015).

Dietary strategies have been investigated as adjunct therapies in RA, including Mediterranean, vegan, vegetarian, elimination, fasting, elemental and ketogenic diets (Philippou et al. 2021). Western dietary patterns, characterised by high intakes of refined carbohydrates, added sugars, saturated fats, omega‐6 fatty acids, and ultra‐processed foods, alongside low fibre and micronutrient density, have been associated with higher inflammatory burden and greater disease activity in RA (Zhang et al. 2025). In contrast, Mediterranean‐style dietary patterns, rich in fibre, polyphenols, and unsaturated fats, have demonstrated improvements in pain, function, and inflammatory markers in most trials (Forsyth et al. 2018; Philippou et al. 2021). Pooled results from a meta‐analysis assessing anti‐inflammatory dietary interventions, compared to usual diets, showed significantly lower RA related pain (Schönenberger et al. 2021).

Reflecting this evidence, the American College of Rheumatology (ACR) 2022 guidelines recommend adherence to a Mediterranean‐style diet and limiting the intake of added sugars, sodium, highly processed foods, refined carbohydrates, and saturated fats. However, the ACR conditionally advises against other formally defined diets, such as paleo, gluten‐free, elimination and vegan diets, due to insufficient evidence supporting their clinical benefit (England et al. 2023). This distinction is relevant given the rapid growth of dietary information accessed through social media and online patient communities.

Surveys of individuals with RA consistently demonstrate strong interest in using dietary modifications to manage symptoms, with 32%–52% reporting dietary changes after diagnosis (Grygielska et al. 2017; Salminen et al. 2002; Tedeschi et al. 2017). In addition, some individuals with RA report that specific foods appear to alleviate or exacerbate their symptoms (Haugen et al. 1991; New et al. 1998; Tanner et al. 1990). One survey reported 36% of people with RA reported using mass media for food‐related guidance after diagnosis (Salminen et al. 2002).

One dietary approach that has gained popularity in online patient communities is the Autoimmune Protocol (AIP) diet, an extension of the Palaeolithic diet. The AIP diet has been promoted by some health professionals, and formal training and certification programmes now exist (AIP Certified 2024). Although no clinical studies have assessed the AIP diet specifically in people with RA, preliminary evidence from three small uncontrolled studies in other autoimmune conditions suggests potential benefits. These include improvements in the quality of life in individuals with Hashimoto's thyroiditis (Abbott et al. 2019; Ihnatowicz et al. 2023), and reductions in clinical symptoms in people with inflammatory bowel disease (Konijeti et al. 2017). Anecdotal reports indicate that some individuals with RA have adopted the AIP diet to manage symptoms, despite the absence of RA‐specific clinical evidence (Laird 2024).

RA is characterised by alterations in the gut microbiome, including reduced microbial diversity and increased abundance of *Prevotella copri*, which have been associated with disease onset and activity (Paolino et al. 2019). Increased intestinal permeability is another recognised feature of RA and is linked to active disease and systemic inflammation (Hecquet et al. 2021; Mucientes et al. 2024). Diet and gut microbial composition can influence intestinal barrier integrity through effects on tight‐junction regulation, microbial metabolites, and immune signalling pathways, providing a biologically plausible link between dietary exposures and autoimmune activity (Brandl et al. 2021).

The AIP diet was initially conceptualised by Professor Loren Cordain (Cordain 1999, 2012) who hypothesised that certain food components, such as lectins, glycoalkaloids, casein, gluten, wheat germ agglutinin, egg white lysozyme, and food additives, may increase intestinal permeability, promote gut inflammation, or act as adjuvants. These effects could facilitate the translocation of dietary and microbial antigens across the gut epithelial barrier into systemic circulation, potentially contributing to autoimmune responses (Cordain 1999; Cordain et al. 2000). These hypotheses were further expanded by biophysicist Sarah Ballantyne, who developed the contemporary AIP diet protocol, providing a detailed rationale and guidance on food exclusions, while emphasising the inclusion of nutrient dense foods, intended to support gut barrier integrity, a healthy gut microbiome and immune regulation (Ballantyne 2013).

The AIP elimination phase excludes grains, legumes, nightshades, dairy, eggs, alcohol, nuts and seeds, sugars, seed oils, and food additives, and emphasises fresh fruits and vegetables, unprocessed meats and seafood, and fermented foods. This elimination phase is typically recommended for one to 4 months, or until symptoms reduce, followed by a structured reintroduction phase to identify individual food sensitivities (Ballantyne 2013). Despite the apparent widespread adoption of the AIP diet, our recent scoping review of elimination‐reintroduction diets in RA identified a paucity of contemporary studies, poorly described dietary protocols and reliance on outdated RA outcome measures, highlighting a significant evidence gap (McNeill et al. 2025).

Given the biological plausibility, patient interest, and absence of RA‐specific clinical evaluation, a pragmatic pilot feasibility study was warranted. The objectives of this study were to evaluate the effect of an AIP elimination diet on self‐reported RA disease activity and selected biometric measures, assess its nutritional adequacy, and determine its acceptability and feasibility in a free‐living context.

## 2 Materials and Methods

### 2.1 Trial Design

**FIGURE 1:**
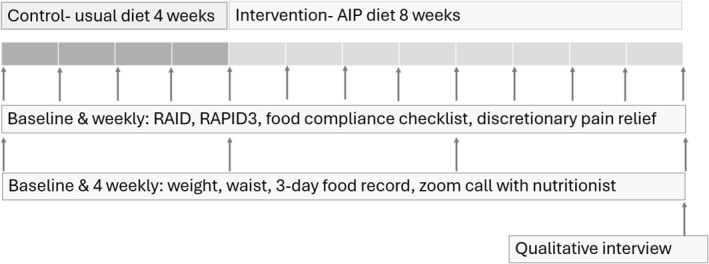
Study design and measures conducted at each time point.

This study was a single‐arm, open‐label feasibility pilot study employing a crossover design, in which participants served as their own controls. During the initial 4‐week control phase, participants maintained their habitual diets. This was followed by an 8‐week intervention phase during which participants adhered to the AIP diet. Quantitative patient‐reported outcome (PRO) data were collected at baseline and weekly for 12 weeks. Biometric data and dietary records were collected at baseline, weeks four, eight and 12. A semi‐structured interview was conducted at the end of the intervention. The study included provisions for remote support, and primary outcome data were collected online in the event of a pandemic‐related lockdown. The overall study structure is illustrated in Figure 1.

### 2.2 Ethics Approval

This study was approved by the Northern B Health and Disability Ethics Committee (HDEC) (21/NTB/55), and by the AUT University Ethics Committee (AUTEC) (21/168). All participants provided written informed consent prior to participation.

## 3 Participants

Between May and August 2021, 11 eligible participants from Auckland, New Zealand were recruited using convenience sampling via social media platforms and Arthritis New Zealand. Inclusion criteria were adults aged 18 years or older, with a clinically confirmed diagnosis of RA of at least six months, stable medication and supplement use for eight weeks, English fluency, computer literacy, access to a computer and smartphone, and the ability to attend four in‐person visits at Auckland University of Technology during the 12‐week study period. Additional diagnoses of rheumatic diseases were not exclusionary criteria in this pilot study.

Exclusion criteria were pregnancy, coexisting health conditions necessitating dietary modifications (e.g., diabetes mellitus, renal impairment, liver disease), or current adherence to a diet excluding any food groups. Interested individuals contacted the primary researcher for eligibility screening. Eligible participants received detailed study information, including procedures, participant responsibilities and consent implications. Each participant commenced the study immediately upon recruitment. All participants continued to receive standard medical care and were advised to continue taking their current supplements.

### 3.1 Intervention Diet

The AIP diet utilised in this study adhered to the guidelines established by Sarah Ballantyne, PhD (Ballantyne 2013), and the AIP Certified Coach programme (Ballantyne et al. 2022). The diet excluded grains, pseudo grains, legumes, dairy, nightshade vegetables and fruits, seeds, nuts, eggs, alcohol, sugar, food additives (e.g., synthetic colours, flavours, emulsifiers, and preservatives), and all processed foods containing these additives (Ballantyne 2013; Cordain 2012). Permitted foods included unprocessed meat, poultry, seafood, all fruits and vegetables (except nightshades), and fats such as avocado, olive, coconut oils, and animal fats. Participants were encouraged to consume omega‐3‐rich seafood, a variety of fruits and vegetables, organ meats, and fermented foods. Alternative flours including cassava, arrowroot, tigernut, green banana, and coconut flour were also permitted. No food was provided; participants purchased their own ingredients and prepared meals independently. There were no restrictions on portion sizes, meal timing, or eating patterns; participants were instructed to eat to satiety. To support adherence, participants received dietary instruction and ongoing support.

### 3.2 Dietary Instruction and Support

During the control phase, participants were instructed to continue their usual diet without modification. At the end of week four, participants attended a one‐hour dietary instruction session led by a registered nutritionist. They received a detailed instruction booklet outlining AIP guidelines, including lists of allowed and restricted foods, meal and snack ideas, recipes, and shopping resources. The booklet also included strategies for navigating potential adherence challenges. Ongoing support was available from the nutritionist via phone or email, as well as through a researcher moderated private Facebook group, offering additional tips, peer support, shared recipes, and Q&A sessions.

### 3.3 Outcomes and Assessments

Quantitative outcomes included PRO questionnaires, biometric measurements, grip strength and dietary assessments. These measures were selected to evaluate the potential physiological and symptomatic effects of the AIP diet, and to examine whether changes in nutrient intake may have contributed to changes in disease activity. Qualitative data were obtained through semi‐structured interviews at the end of the study to explore participants' experiences following a restrictive dietary protocol. Qualitative data analysis and results are reported separately.

### 3.4 Primary Outcomes

#### 3.4.1 Patient Reported Outcome (PRO) Questionnaires for RA

The primary endpoints were changes in disease activity and RA relevant symptom domains at week eight. These measures were assessed using self‐administered PRO questionnaires collected weekly via the QuestionPro platform (QuestionPro Inc.). Two validated tools were employed: Routine Assessment of Patient Index Data 3, (RAPID3), which assesses disease activity, and correlates significantly with the Disease Activity Score‐28 (DAS28), Clinical Disease Activity Index (CDAI), Simplified Disease Activity Index (SDAI), and DAS28‐C‐reactive protein (CRP) scores (Bergman et al. 2022; Berthelot 2014; Pincus, Furer, et al. 2011). The RA Impact of Disease (RAID) developed by the European League Against Rheumatism (EULAR), assesses seven domains considered most important by patients: pain, physical function, fatigue, sleep, physical well‐being, emotional well‐being, and coping (Gossec et al. 2011; Radawski et al. 2019). RAID scores correlate well with total DAS28‐CRP or DAS28‐erythrocyte sedimentation rate (ESR) (Mistry et al. 2020). Both RAPID3 and RAID were scored from 0 to 10, with higher scores indicating greater disease activity. Weekly reports also included secondary endpoints, encompassing statements regarding any positive or negative health effects attributed to diet and documentation of daily use of discretionary anti‐inflammatory or pain relief medication.

### 3.5 Secondary Outcomes

#### 3.5.1 Biometric Data

Secondary endpoints assessed changes in biometric measures. Data were collected at baseline, weeks four, eight and 12. Measurements included weight, body mass index (BMI), body composition (assessed using Tanita bioelectrical impedance analysis [BIA] scales), waist circumference (cm), bilateral grip strength (measured using a dynamometer [kg]), and blood pressure (mmHg). Waist circumference was measured at the navel over bare skin or light clothing, and weight was recorded at a consistent time of day.

Due to COVID‐19 lockdown restrictions implemented on 17 August 2021 for 3.5 months, in‐person data collection was discontinued. Consequently, BIA, blood pressure and grip strength measures were unable to be collected during or post‐intervention. Participants were instructed to self‐record their waist circumference and weight using a tape measure and home scales, maintaining consistency in the time of day and clothing worn. All subsequent support and communication were conducted remotely, via email, phone, or video conferencing.

#### 3.5.2 Dietary Assessment and Adherence

Participants completed 3‐day dietary records at baseline and week four (control phase), and weeks eight and 12 (AIP phase) using the ‘Easy Diet Diary’ app (Xyris Software). To improve accuracy, participants photographed all meals and snacks using either the Easy Diet Diary or ‘See What You Eat’ app (Health Revolution Ltd.). Dietary data were analysed using FoodWorks 10 (Xyris Software). A daily adherence checklist was completed by participants to track the consumption of non‐compliant foods, and these checklists were submitted weekly. Nutritional analysis findings are reported in a separate manuscript.

Weekly participant feedback on the ease of implementing the AIP diet and perceived changes in food costs compared to their usual diet was collected using a five‐point Likert scale response to a statement.

### 3.6 Data Analysis

#### 3.6.1 Quantitative Analysis

Given the exploratory nature of this study and the small sample size, quantitative data were analysed and reported as changes in individual responses. Data collected via QuestionPro were downloaded and processed using online scoring calculators for RAPID3 and RAID outcomes. RAPID3 scores were calculated using RheumGuide (Omar and Pincus 2019). RA disease activity categories are defined as follows: remission ≤ 1.0, low activity > 1.0–2.0, moderate activity > 2.0–4.0, and high activity > 4.0–10. Treatment responses were categorised according to validated thresholds (Pincus, Hines, et al. 2011):

- Good response: reduction > 1.2/10 with an endpoint of < 2/10
- Moderate response: reduction > 1.2/10 with an endpoint ≥ 2/10, or a reduction of 0.6–1.2 with an endpoint ≤ 4
- Poor response: reduction < 0.6/10 or a reduction of 0.6–1.2 with an endpoint > 4

RAID scores were calculated using RheumCalc (2021). RAID categories were defined as follows: remission (≤ 3), low impact (> 3 to ≤ 4), moderate impact (> 4 to ≤ 6), or severe impact (> 6) (Rodríguez Esquíroz et al. 2024). A score of ≤ 2 is considered a patient acceptable status. A minimum clinically important improvement (MCII) was defined as an absolute change of 3 points or a relative change of 50% (Dougados et al. 2012). Exploratory paired comparisons were conducted for RAPID3 and RAID, using individual change scores calculated as the week 8 AIP value minus the mean score across the 4‐week control phase. Change‐score distributions were approximately normal, so mean change scores were compared against zero using one‐sample *t*‐tests. Results are presented as mean differences, effect sizes (Cohen's *d*), and 95% confidence intervals. While *p*‐values are reported, they should be interpreted with caution given the exploratory nature of the analysis and the small sample size.

Three‐day dietary records were analysed to quantify daily servings per food group and compare these between habitual and AIP diets.

Discretionary pain relief data were collected, and the doses of non‐steroidal anti‐inflammatory drugs (NSAIDs) and paracetamol per week were compared between the usual diet phase (mean of 4 weeks) and the intervention endpoint.

## 4 Results

### 4.1 Intervention Uptake

Of the 21 individuals who expressed interest in the study, 11 eligible participants were recruited. Participants commenced the study on enrolment between June 25 and August 12, 2021. One participant withdrew after the 4‐week control phase because of difficulties adhering to the protocol during a COVID‐19 lockdown. The reserve participant was subsequently enroled on August 31, 2021. Another participant withdrew at the end of the 4‐week control phase due to a broken ankle. A total of 11 participants completed the control phase, and nine completed the full 12‐week study. Table 1 presents baseline demographic data for the nine participants who completed the study, including age, gender, ethnicity, antibody status, comorbidities, RA duration, current medications for RA, and supplements.

**TABLE 1:** Baseline characteristics of 9 participants who completed the study.

Gender, age 2021	Ethnicity	BMI baseline	Years since RA diagnosis	Antibodies	Comorbidities	RA medication	Supplements
1. F, 46	NZ European	23.5	19	Anti‐CCP + ve RF + ve	Raynaud's	Methotrexate	Folic acid Collagen
2. F, 60	South African European	26.8	5	Anti‐CCP + ve	Secondary Sjogren's	Hydroxychloroquine Methotrexate	Folic acid
3. M, 50	NZ European	24.1	2	Anti‐CCP + ve RF + ve	Rust tree allergy	Hydroxychloroquine Methotrexate	Folic acid Zinc, Omega3, probiotic, B12, Vit D, Vit K2
4. F, 49	Irish European	31.1	1	Seronegative ANA 320	Meniere's Rosacea Fibromyalgia	Methotrexate Hydroxychloroquine	Folic acid Magnesium, multivitamin
5. M, 63	NZ European	26	3	RF + ve Anti‐CCP + ve	None	Methotrexate Adalimumab	Folic acid
6. F, 60	NZ European	31.4	7	Anti‐CCP + ve RF + ve	Idiopathic neutropenia Migraine Osteoarthritis	Methotrexate	Folic acid Omega3, turmeric Glucosamine/chondroitin, probiotic
7. F, 40	Canadian European	29.9	7	RF + ve Anti‐CCP‐ve	Dysautonomia	Methotrexate	Folic acid Zinc, turmeric, magnesium, vitamin C
10. F, 59	NZ European	20.3	23	Seropositive	Sjogren's IBS AI connective tissue: Rhupus Mild neutropenia	Hydroxychloroquine Leflunomide Etanercept	None
11. F, 43	NZ European	23.2	13	Anti‐CCP + ve ANA 1:640	Reflux, hypersensitive oesophagus Raynauds	Rituximab	Collagen, vit C, magnesium

*Note:* Participants 8 and 9 withdrew after the usual diet phase and are not included.

Abbreviations: ANA, Anti‐nuclear antibody; Anti‐CCP, Anti‐Cyclic Citrullinated Peptide; F, female; M, male; NZ, New Zealand; RF, rheumatoid Factor.

### 4.2 Effect of AIP Diet Intervention on RAPID3 Scores

**FIGURE 2:**
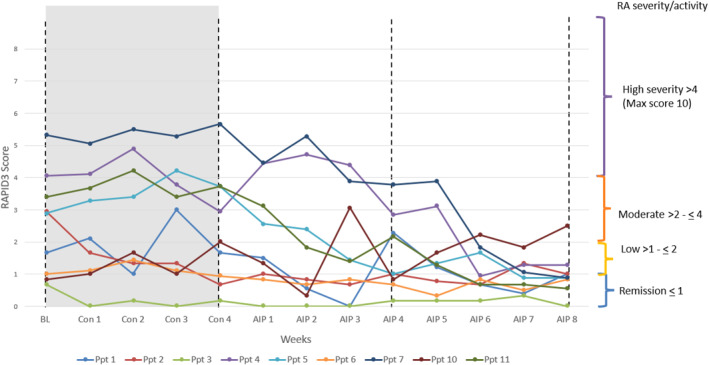
Weekly RAPID3 scores for individual participants. AIP, Autoimmune protocol diet intervention, 8 weeks; Con, control, baseline to end week 4, no dietary changes; Ppt, participant.

RAPID3 scores generally showed progressive improvement during the AIP phase, with the most notable reductions in RA activity observed by weeks 6–8 (Figure 2).

Four participants demonstrated a clinically meaningful reduction in RAPID3, while one participant met the criteria for a moderate response (Table 2). Two participants transitioned from low disease activity to remission, and one remained with low disease activity throughout the 12‐week period. One participant had an increase in RAPID3 during the AIP intervention, moving from low to moderate disease activity.

**TABLE 2:** Individual changes in RAPID3 scores: Comparison between control phase average and week eight of the AIP intervention.

Participant number	Control: Mean of baseline to end week 4	End week 8 AIP	Change baseline to end AIP	Baseline RAPID3 level (4‐week mean)	Week 8 AIP RAPID3	Clinical relevance of change in activity level
Ppt 1	1.89	1	−0.89	Low	Remission	Moderate
Ppt 2	1.588	1	−0.588	Low	Remission	
Ppt 3	0.202	0	−0.202	Remission	Remission	No change
Ppt 4	3.956	1.28	−2.676	Moderate	Low	Good
Ppt 5	3.5	0.89	−2.61	Moderate	Remission	Good
Ppt 6	1.12	0.83	−0.29	Low	Remission	
Ppt 7	5.368	0.89	−4.478	High	Remission	Good
Ppt 10	1.3	2.5	+1.2	Low	Moderate	Worse
Ppt 11	3.678	0.56	−3.118	Moderate	Remission	Good

### 4.3 Effect of AIP Diet Intervention on RAID Scores

**FIGURE 3:**
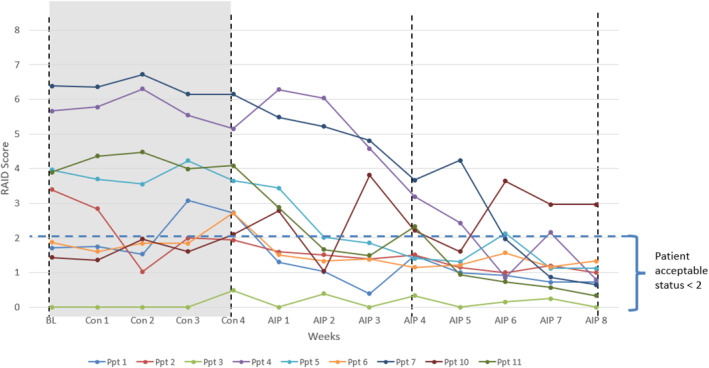
Weekly RAID scores for individual participants. AIP, Autoimmune protocol diet intervention, 8 weeks; Con, control, baseline to end week 4, no dietary changes.

RAID scores improved during the AIP phase for seven of the nine participants (Table 3). Three participants demonstrated an absolute reduction of > 3 points, while six demonstrated a relative reduction of > 50%. By week eight of the AIP intervention, seven of nine participants had RAID scores below 1.5, indicating a patient‐acceptable symptom state (≤ 2). Similar to the RAPID3 scores, the greatest improvements were observed between weeks six and eight of the AIP phase (Figure 3). One participant who did not respond to the intervention experienced a 1.3‐point increase, resulting in a final score of 2.96. Another participant, who began the study with low disease activity maintained stability throughout.

**TABLE 3:** Baseline and endpoint RAID scores, with relative and absolute change values, for individual participants.

Participant number	Control mean of baseline to end week 4	AIP end week 8	Absolute change	Relative change
Ppt 1	2.16	0.72	−1.44	−67%
Ppt 2	2.24	1	−1.24	−55%
Ppt 3	0.1	0	−0.1	0
Ppt 4	5.69	0.8	−4.89	−86%
Ppt 5	3.82	1.12	−2.7	−71%
Ppt 6	1.97	1.33	−0.64	−32%
Ppt 7	6.35	0.64	−5.71	−90%
Ppt 10	1.69	2.96	+1.27	+75%
Ppt 11	4.16	0.33	−3.83	−92%

### 4.4 Exploratory Inferential Analysis of RAPID3 and RAID Scores

A summary of paired *t*‐test results, including mean change scores, 95% confidence intervals, and effect sizes, is presented in Table 4. At a group level, there were reductions in both RAPID3 and RAID scores from the control‐phase mean to the week‐8 intervention endpoint (RAPID3: mean change = −1.52, 95% CI [−2.90, −0.14], *p* = 0.035, Cohen's *d* = −0.85; RAID: mean change = −2.14, 95% CI [−3.92, −0.36], *p* = 0.024, Cohen's *d* = −0.93). Although statistically significant, these analyses were exploratory and are presented to complement the descriptive findings.

**TABLE 4:** Paired *t*‐test results for changes in RAPID3 and RAID scores (*n* = 9).

	Mean change	95% CI (mean change)	*t* (8)	*p*	Cohen's *d*	95% CI (Cohen's *d*)
RAPID3 change	−1.52	[−2.90, −0.14]	−2.54	0.035	−0.85	[−1.60, −0.06]
RAID change	−2.14	[−3.92, −0.36]	−2.78	0.024	−0.93	[−1.70, −0.12]

*Note:* Negative values indicate improvement relative to the control‐phase mean.

### 4.5 Fatigue and Sleep

Seven of the nine participants showed meaningful improvements in fatigue and four in the sleep scales of RAID. The mean fatigue scores decreased from 4.44/10 (range 0–10) at baseline to 1.11 (range 0–4) at week 12. Eight of nine participants reported a final score of 0 or 1. Participant 4, who also had a fibromyalgia diagnosis, reported a substantial reduction in fatigue from 10 to 1. One participant reported an increase in fatigue during the study. The mean sleep score decreased from 3.77/10 (range 0–10) at baseline to 1.22 (range 0–2) by week 12 [Supporting Information S1: Figures 1 and 2].

### 4.6 Discretionary Pain Relief Use

The weekly mean discretionary NSAID use during the usual diet phase (mean of 4 weeks) was 1.97 doses per week (range 1–6.75), and at the endpoint (week eight of the AIP phase), the mean was 0.11 doses per week (range 0–1). The weekly paracetamol use during the usual diet phase (mean weeks 1–4) was 1.78 doses per week (range 0–5.75), and at the end point (week eight of the AIP phase) it was 2.22 (range 0–12) doses per week [Supporting Information S1: Table S2].

### 4.7 Dietary Adherence

Reported dietary infractions were minimal [Supporting Information S1: Table S1] and typically consisted of minor ingredients in otherwise compliant foods or occasional deviations in one or two meals per week. The impact of these minor infractions on outcomes is difficult to determine; however, most participants who reported occasional infractions nonetheless demonstrated improvements in disease activity, with only one report of an acute increase in RA symptoms following a specific food exposure.

### 4.8 Changes in Food Group Intake

During the AIP phase, no participants reported consuming foods classified as ‘not allowed’ (eggs, grains, nuts &seeds, legumes, nightshades, dairy, and alcohol) in their food diaries. Changes in median daily servings from the habitual diet to the AIP phase (calculated from the median of individual participant means across both 3‐day records) included an increase in seafood (from a median of 0.3–0.8 servings/day), fruit (from 1 to 2.9 servings/day), and vegetable intake (from 4.5 to 11.5 servings/day) (Table 5). Refined cereal grain intake, which averaged 5 servings per day during the control phase, decreased to zero during the AIP phase. Added sugar intake reduced from 31 to 17 g per day. These changes in plant food consumption resulted in an increase in the median fibre intake from 25.5 to 35.6 g/day. Conversely, red meat consumption increased from a median of 0.6 per day to 0.9 servings per day, two participants exceeded the recommended intake during the control phase, and five participants exceeded it during the AIP phase.

**TABLE 5:** Intake of foods by group, from 4‐weekly, 3‐day food records, median (range).

Food groups	Baseline, Con	Week 4, Con	Week 8, AIP	Week 12, AIP	NZ recommended servings/day
Fruit	1.1 (0.3–1.8)	0.6 (0–1.9)	3.1 (1.6–8.8)	3.5 (2–6.9)	2
Vegetables	4.7 (1.4–5.4)	5.9 (2.9–14.6)	13.0 (4.8–22.3)	11 (5.1–24)	Males: 51–70, 5.5 Females: 5
Grains	5.7 (4–8.5)	6.7 (1.2–8.7)	0 (0–2.2)	0.3 (0–2.2)^a^	Whole or high fibre Males: 19–70, 6 Females: 19–50, 6 Females: 51–70, 4
Wholegrains	0.9 (0–3.4)	0.9 (0–1.4)	0	0	
Nuts, seeds	0.3 (0–1.3)	0.3 (0–0.5)	0	0	*Combined per day*: Males: 51–70, 2.5 Females: 19–50, 2.5 Females: 51–70, 2 *Red meat, limit to*: < 500 g cooked, 700–750 g raw/week
Poultry	0.7 (0–1.4)	0.7 (0–2.2)	0.9 (0.5–3.1)	0.8 (0–1.8)	
Seafood	0.1 (0–0.8)	0.3 (0–1.5)	0.8 (0–2.3)	0.7 (0–2.2)	
Eggs	0.3 (0.1–1.8)	0.1 (0–1.8)	0	0	
Red meat	0.7 (0–1.6)	0.6 (0–1.2)	0.4 (0–3.4)	1.3 (0.6–6.06)	
Processed meat	0.2 (0–1.8)	0.2 (0–0.3)	0 (0–0.7)^b^	0 (0–0.7)^b^	Limit intake
Cheese^c^	0.2 (0–1.2)	0.4 (0–1.3)	0	0	Dairy, reduced fat, Males: 51–70, 2.5 Females: < 51, 2.5 Females: > 50, 4
Fibre g: Male	22.8 (16.5–29)	25.2 (13.8–36.6	50.9 (40.7–61)	41.2 (30.6–51.7)	Males: 30 g
Female	22.8 (12.7–25.6)	25.8 (14.2–32.9)	39.5 (23.5–52.7)	33.1 (21.4–58.2)	Females: 25 g
Added sugar g	23.8 (15.2–170.3	24.7 (10–57.7)	5.1 (1.7–45.3)	13 (0.7–35.2)	NZ: ≤ 25 g, WHO: ≤ 50 g
Alcohol	0.3 (0–5.5)	0.3 (0–2.9)	0	0	Females: 2 max/day, 10/week Males: 3 max/day, 15/week

*Note:* Grey shaded columns are the usual diet phase.

^a^ Tapioca starch, not a grain.

^b^ Bacon no additives.

^c^ Other dairy: Milk yoghurt not able to be assessed.

### 4.9 Effect of the AIP Intervention on Weight, BMI and Waist Circumference

**FIGURE 4:**
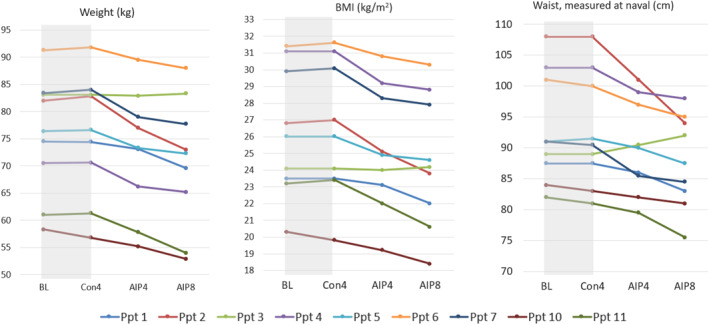
Changes in weight, BMI, and waist circumference for individual participants.

BMI remained stable during the control phase (mean 26.3, range 20.1–31.1) and decreased by the end of the AIP phase (mean 24.5, range 18.4–30.3) (Figure 4). Eight participants lost weight during the AIP phase, with a mean loss of 5 kg (range +0.2 to −9.8 kg). There was a mean reduction in waist circumference of 4.8 cm (range +3.0 to −14 cm).

### 4.10 Adverse Events and Unintended Consequences

During the initial one to 2 weeks of the AIP diet, six participants reported intermittent headaches, and during weeks one to four, seven reported digestive symptoms, including increased bloating. These symptoms did not persist. However, two participants experienced persistent adverse effects during the AIP intervention: one reported regular diarrhoea beginning in week five, and another reported lethargy and a loss of appetite, resulting in unintended weight loss that persisted over the 8 weeks. Supporting Information S1: Table S3 summarises the positive and negative effects attributed to the diet, as documented in weekly participant feedback.

### 4.11 Costs and Ease of Implementation of AIP Diet

**FIGURE 5:**
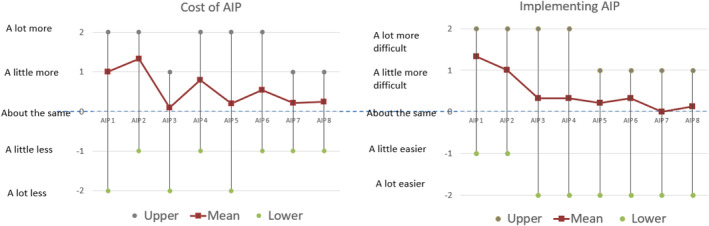
Food costs and ease of implementation of AIP diet compared to habitual diet, weeks 5–12, AIP phase.

Participants generally reported higher food expenses at the beginning of the intervention, primarily due to the need to purchase AIP‐compliant pantry staples (Figure 5). However, some participants who frequently dined out reported reduced overall spending.

## 5 Discussion

This 12‐week pilot study assessed the impact of the AIP diet on PROs and dietary intake in individuals with RA. To our knowledge, this is the first clinical investigation of the feasibility and potential effects of the AIP diet in people with RA. Adherence during the 8‐week intervention was high, with minimal reported infractions.

The primary outcome measures, RAPID3 and RAID scores, demonstrated clinically meaningful improvements for most participants. Four achieved a good RAPID3 response, and three transitioned from low disease activity to remission. Regarding RAID scores, three participants achieved an absolute reduction exceeding three points, while three others achieved a relative change greater than 50%. Mean discretionary NSAID use decreased during the AIP phase. Collectively, these findings suggest that the AIP diet may be associated with improvements in patient reported symptoms in some individuals with RA, warranting evaluation in controlled studies. However, approximately half of the participants entered the study with low baseline disease activity, which may limit generalisability to individuals with moderate‐to‐high disease activity.

Fatigue and sleep domains improved during the AIP phase. Similar findings have been reported in dietary interventions using related elimination‐based protocols. A modified Palaeolithic diet in individuals with multiple sclerosis demonstrated greater reductions in fatigue, as measured by the Modified Fatigue Impact Scale, compared to both a medium‐chain triglyceride‐based ketogenic diet (*p* = 0.02) and the Swank low‐fat diet (*p* ≤ 0.05) (Lee et al. 2021; Wahls et al. 2021). In RA, the anti‐inflammatory ITIS diet (based on Mediterranean principles, and excluding dairy, gluten, processed foods, alcohol, nightshades, and red meat) reported a ≥ 50% reduction in fatigue in nine participants, and ≥ 50% reduction in pain in seven following a 2‐week intervention in 20 individuals with RA (Coras et al. 2021). Given the lack of effective pharmacological approaches for managing fatigue in people with RA, the impact of diet on RA fatigue across both the ITIS and AIP protocols warrants further investigation. Although current evidence is encouraging, it remains insufficient to support dietary interventions as a treatment for RA‐related fatigue (Dures et al. 2024).

Sleep, an often‐overlooked domain of RA management (Mustafa et al. 2019; Taylor et al. 2016), was associated with improvements in this study. Sleep is recognised as one of the top five concerns for people with RA (Nowell et al. 2021), and is recommended as an outcome measure in intervention studies (Taylor et al. 2021). To our knowledge, no dietary intervention studies in RA have assessed sleep as an outcome. However, a cross‐sectional study of 205 individuals with RA found that higher adherence to a Mediterranean diet was associated with better sleep quality and lower RAID scores (*p* < 0.05) (Ingegnoli et al. 2020).

### 5.1 Possible Dietary Mechanisms

The mechanisms underlying improvements in PROs are likely multifactorial and can broadly be grouped into three overlapping domains: (1) improved dietary quality, (2) removal of potential trigger foods, and (3) microbiome modulation.

#### 5.1.1 Dietary Quality and Food Exclusions

The AIP diet promotes the consumption of nutrient‐dense foods with anti‐inflammatory qualities, overlapping with Mediterranean dietary patterns that are associated with reduced RA disease activity, particularly in highly adherent individuals (Raad et al. 2024; Vadell et al. 2020). In the present study, intake of fruit, vegetables, olive oil, avocado and seafood increased. These foods provide fibre, polyphenols, long‐chain omega‐3 fatty acids and monounsaturated fats (MUFAs), which influence inflammatory pathways (Oliviero et al. 2015). Previous cross‐sectional research in people with RA found that consuming fish more than twice a week was associated with significantly lower DAS28‐CRP scores compared with consumption once per month, with each additional weekly serving associated with a 0.18 point reduction in DAS28‐CRP (Tedeschi et al. 2018). Extra virgin olive oil (EVOO) contains both oleic acid and phenolic compounds that modulate inflammation (Lucas et al. 2011) and the combination of EVOO with omega‐3 supplementation improved patient global assessments compared to omega‐3 alone (Berbert et al. 2005).

The AIP diet excludes multiple food groups, some of which have been reported to provoke symptoms in subsets of individuals in elimination‐reintroduction studies (Cooper 1991; Gamlin and Brostoff 1997) and case reports, and includes gluten (Bruzzese et al. 2021; Isasi et al. 2016; Parke and Hughes 1981), corn (Williams 1981), dairy (Panush et al. 1986; Ratner et al. 1985), and nightshades (Denton 2012). Because dietary quality improves during an elimination diet, isolating independent effects is challenging. However, controlled studies suggest that targeted exclusions may confer additional benefits in some individuals. A randomised controlled trial (RCT) comparing the ITIS diet with a Mediterranean diet (*n* = 44) found similar reductions in CDAI at 3 months, however only the ITIS group achieved significant improvements in pain and fatigue. Remission occurred in 11.1% of ITIS participants compared with none in the Mediterranean diet group (Sala Climent et al. 2023). Likewise, a Mediterranean diet study (*n* = 40) excluding dairy and gluten improved quality of life, pain and CRP compared to a matched diet without exclusions (Guagnano et al. 2021).

#### 5.1.2 Gut Health and Microbiome Modulation

Participants markedly increased fibre, fruit and vegetable intake, while eliminating refined grains and reducing added sugars, shifting towards a whole‐food carbohydrate profile. These changes influence gut microbiota composition, short‐chain fatty acid (SCFA) production and intestinal permeability, pathways implicated in RA pathophysiology (Attur et al. 2022; Guerreiro et al. 2018). In a 28‐day intervention in RA, adding a multi‐fibre supplement alone (with no other dietary changes) improved SF‐36 physical functioning and reduced serum zonulin and serum calprotectin (markers of intestinal permeability and inflammation) (Häger et al. 2019). This fibre intervention also increased SCFA and reduced pro‐inflammatory cytokines within 15 days (Dürholz et al. 2020). In a study in older adults, adding three servings of polyphenol‐rich food a day increased beneficial fibre‐fermenting and butyrate‐producing bacteria, improved intestinal permeability, and reduced serum zonulin levels (Del Bo’ et al. 2021). Increased microbial diversity has also been observed in Italians consuming a Paleolithic diet (which has a similar carbohydrate profile to AIP) in a modern context (Barone et al. 2019). Responders in the ITIS study demonstrated favourable shifts in gut microbiota (Coras et al. 2020, 2021). Although biomarkers were not measured in this study, gut‐mediated pathways provide biological plausibility for the improvements observed with AIP.

### 5.2 Weight Loss and Satiety

Weight loss was not an intended outcome; however, all but one participant lost weight and reported increased satiety. This likely reflects replacement of refined carbohydrates with higher‐fibre, lower energy density fruits and vegetables, and increased protein intake. Palaeolithic‐style diets, while less restrictive than the AIP diet, share a similar emphasis on fruit, vegetables and animal protein, promote greater satiety and reduce energy intake compared with a diet for type 2 diabetes or a Mediterranean diet (Jönsson et al. 2010, 2013). Other ad libitum Paleo diet interventions have also reported spontaneous reductions in energy intake and body weight, as well as reduced cravings for sugary foods (Genoni et al. 2016; Österdahl et al. 2008; Ryberg et al. 2013).

### 5.3 Adverse Effects

Most participants reported transient headaches and gastrointestinal symptoms during dietary transition; however, two reported persistent adverse effects. One reported a flare of RA symptoms, persistent loss of appetite, and unintended weight loss, and another developed diarrhoea from week five onward. Causality is uncertain; however, dietary factors warrant consideration. Participant 10 consumed daily cassava‐based products, which have been anecdotally associated with symptom exacerbation in some individuals following AIP (AIP Diet Support Group 2026), and discontinuation of habitual steel‐cut oats removed a prebiotic fibre source (Van den Abbeele et al. 2018). The diarrhoea in one participant may reflect the substantial increase in fruit, vegetables and fibre during the AIP phase (fruit 1.4 to 7.9, vegetables 7 to 16.6 servings/d, fibre 33–56 g/d). This is consistent with a report of diarrhoea in five of 22 participants in a Palaeolithic diet study in Australia (Genoni et al. 2016). However, similar adverse effects have not been reported in other AIP studies (Abbott et al. 2019; Ihnatowicz et al. 2023; Konijeti et al. 2017).

### 5.4 Limitations

This study has several limitations. The small sample size and absence of a control group limit generalisability. Participants were self‐selected, willing to undertake an intensive dietary intervention, and highly motivated, potentially limiting the applicability of the results to the broader RA population. Much of the intervention period coincided with a prolonged pandemic lockdown, which may have influenced adherence and reduced exposure to social eating situations that typically challenge compliance in real‐world settings.

Outcome assessments, RAPID3 and RAID, although validated and clinically meaningful, rely on self‐reports, which may limit the strength of conclusions in the absence of objective inflammatory markers or clinician‐assessed disease activity. Low baseline disease activity limits inference regarding individuals with higher disease activity. The absence of inflammatory or microbiome biomarkers limits the mechanistic interpretation of observed symptom changes.

Self‐measured biometrics (weight and waist circumference) may lack precision compared with clinic‐based assessments. In addition, body composition (BIA) and hand‐grip strength could not be collected due to lockdown restrictions, limiting the ability to distinguish between fat mass and lean mass changes. This is particularly relevant given the risk of sarcopenia or cachexia in RA populations.

Finally, online support from the researcher may be a key aspect influencing high adherence or diet logging behaviours. Future studies may not have sufficient resources to replicate this level of support, which limits feasibility in routine clinical settings. Following the study, participants managed the food re‐introduction process independently. A 1‐year follow‐up study was conducted, and results will be reported separately.

### 5.5 Recommendations for Future Research

Future research should employ appropriately powered RCT designs with blinded outcome assessments. Recruitment criteria should include a minimum baseline disease activity threshold to enhance the ability to detect clinically meaningful changes. The inclusion of objective outcome measures, including inflammatory markers, gut health biomarkers, and clinically assessed disease activity, would strengthen efficacy evaluation and provide mechanistic insights. Body composition and functional measures would assess potential risks related to lean mass loss.

Longer follow‐up periods and structured reintroduction are needed to assess sustainability and safety and identify specific dietary triggers. To address potential confounding factors by dietary quality, comparator groups with similar overall dietary quality, differing primarily by food exclusions are recommended. Given the restrictive nature of the AIP diet, further investigation is warranted to determine whether all eliminations are necessary to achieve clinical benefit.

## 6 Conclusions

This pilot feasibility study provides preliminary evidence that the AIP dietary intervention was associated with improvements in patient‐reported outcomes of disease activity, pain, fatigue, and sleep in some individuals with RA. Individual responses varied, and a small number of participants reported adverse effects or no improvement. Given the small sample size, low baseline disease activity, and lack of a control group, the findings should be interpreted as hypothesis‐generating.

Larger controlled trials incorporating participants with moderate or higher disease activity, objective biomarkers, structured reintroduction protocols, and longer‐term follow‐up are needed to assess the diet's safety, efficacy and sustainability. Given the restrictive nature of the AIP diet, further research is needed to identify the components contributing to clinical benefit and to determine which eliminations are necessary and which dietary components should be prioritised.

## Author Contributions

J.M., C.Z., G.M., and R.G. contributed to conceptualisation and study methodology. J.M. conducted the study, collected and analysed the data, and drafted the manuscript. R.G., C.Z. and G.M. reviewed the data and analysis. C.Z., G.M., and R.G. contributed to the critical revision and editing of the manuscript. All authors approved the final version.

## Funding

The authors have nothing to report.

## Ethics Statement

This study was approved by the Northern B Health and Disability Ethics Committee (HDEC) (21/NTB/55), and by the Auckland University of Technology Ethics Committee (AUTEC) (21/168).

## Consent

All participants provided written informed consent prior to participation.

## Conflicts of Interest

The authors declare no conflicts of interest.

## Supporting information


Supporting Information S1

## Data Availability

The data that support the findings of this study are available from the corresponding author upon reasonable request.
