# Supplementary material for: The Effect of an Autoimmune Protocol (AIP) Diet in Adults With Rheumatoid Arthritis: A Single Arm Crossover Pilot Feasibility Study
Source: Musculoskeletal Care. 2026 Apr 4;24(2):e70214. doi: 10.1002/msc.70214 (PMC13049695; doi:10.1002/msc.70214)

**Supplementary information for:**

**The effect of an autoimmune protocol (AIP) diet in adults with rheumatoid arthritis. A single arm crossover pilot feasibility study**

Julianne McNeill, Gael Mearns, Rebecca Grainger, Caryn Zinn

**Table S1:** All dietary infractions recorded by participants on their weekly checklists

**Table S2:** Discretionary pain relief use

**Table S3:** Summary of positive and negative effects reported associated with diet

**Figure S1:** Weekly individual fatigue scores over 12 weeks

**Figure S2:**  Individual weekly sleep scores over 12 weeks

**Table S1: Dietary adherence**

Reported dietary infractions were minimal (Table S1), typically consisting of minor ingredients in otherwise compliant foods or occasional deviations in one or two meals per week.

**Table S1** All dietary infractions recorded by participants on their weekly checklists.

| Participant | AIP week 1 | AIP wk 2 | AIP wk 3 | AIP wk 4 | AIP wk 5 | AIP wk 6 | AIP wk 7 | AIP wk 8 |
| --- | --- | --- | --- | --- | --- | --- | --- | --- |
| 1 | yeast extract x2, xylitol x 1, colour x 1 | 0 | E220 x 2 | E220x1, 466, 415, 436 x1 | 0 | cayenne pepper x1 | cornstarch x 2 | 0 |
| 2 | 0 | 0 | 0 | 0 | 0 | 0 | potato accidental | 0 |
| 3 | 0 | 0 | 0 | green peas | 0 | 0 | 0 | 0 |
| 4 | choc fish x1, choc twirl x1 | agave syrup 5ml, 2 x fries | M&Mx5, 1/2 choc biscuit | 0 | sip red wine, 2 square chocolate, cayenne pepper, 1/2 choc biscuit | small piece garlic bread, 2 fries, 4 squares chocolate | GF pizza, fries | hamburger + cheese |
| 5 | 0 | 0 | 0 | 0 | 0 | 0 | 0 | 0 |
| 6 | guar gum x1, erythritol x2 E202x1 (additives in foods) | Erythritol x 3, E202x1 | raw macaroon with cocoa butter x1, e202 x1 | e202x1 | 0 | 0 | e202x1,  wheat coating x1, sunflower oil x1 | e202x1 |
| 7 | 0 | 0 | 0 | 0 | 0 | 0 | 0 | 0 |
| 10 | Beans x1, stevia x1, pepper x1 | Additive x1 | Cayenne x1  spice mix x1 | Stevia x2 | maple syrup flavour x1 | instant gravy x1 | stevia x2 |  |
| 11 | 0 | 0 | 0 | 0 | 0 | 0 | 0 | 0 |

E220: Sulphur dioxide, E466: sodium carboxymethyl cellulose, E415: xanthan gum, E436: Polyoxymethylene sorbitan tristearate, E202: Potassium sorbate

**Table S2: Discretionary pain relief use**

Participants took either an NSAID or paracetamol or both

**A standard dose for a participant was counted as 1 dose, as follows:**

NSAID use: Ibuprofen 200 or 400mg, Diclofenac 75mg, Celecoxib 100 or 200mg,

Paracetamol: 500mg (taken as 2x500mg = 1 gram dose) or Panadol osteo 665mg, 2 tabs = 1 dose

Combined (Nuromol) Ibuprofen 200, paracetamol 500 (taken as 2 caps = Ibuprofen 400mg + 1gram paracetamol)

**Table S2:** Discretionary pain relief use

|  | | NSAID doses per week | | |  |  | |  | |  | | |  | |  | |  | |  | |  | |
| --- | --- | --- | --- | --- | --- | --- | --- | --- | --- | --- | --- | --- | --- | --- | --- | --- | --- | --- | --- | --- | --- | --- |
|  | Con1 | | Con2 | Con3 | | | Con4 | | AIP1 | | AIP2 | AIP3 | | AIP4 | | AIP5 | | AIP6 | | AIP7 | | AIP8 |
| Ppt 1 | 2 | | 1 | 2 | | | 1 | | 2 | | 0 |  | |  | | 0 | | 0 | | 0 | | 0 |
| Ppt 2 | 0 | | 0 | 0 | | | 0 | | 0 | | 0 | 0 | | 0 | | 0 | | 0 | | 0 | | 0 |
| Ppt 3 | 0 | | 0 | 0 | | | 0 | | 0 | | 0 | 0 | | 0 | | 0 | | 0 | | 0 | | 0 |
| Ppt 4 | 1 | | 2 | 2 | | | 1 | | 4 | | 2 | 2 | | 1 | | 1 | | 1 | | 0 | | 0 |
| Ppt 5 | 0 | | 4 | 2 | | | 2 | | 0 | | 0 | 0 | | 0 | | 0 | | 0 | | 0 | | 0 |
| Ppt 6 | 5 | | 4 | 1 | | | 4 | | 0 | | 0 | 0 | | 2 | | 0 | | 1 | | 2 | | 0 |
| Ppt 7 | 3 | | 3 | 2 | | | 2 | | 3 | | 2 | 2 | | 2 | | 2 | | 1 | | 1 | | 1 |
| Ppt 10 | 0 | | 0 | 0 | | | 0 | | 0 | | 0 | 0 | | 0 | | 0 | | 0 | | 0 | | 0 |
| Ppt 11 | 2 | | 3 | 10 | | | 12 | | 2 | | 1 | 1 | | 0 | | 2 | | 0 | | 0 | | 0 |
| Mean | 1.44 | | 1.89 | 2.11 | | | 2.44 | | 1.22 | | 0.56 | 0.63 | | 0.63 | | 0.56 | | 0.33 | | 0.33 | | 0.11 |
| Mean usual diet |  | |  |  | | | 1.97 | |  | |  |  | |  | |  | |  | |  | |  |
|  |  | |  |  | | |  | |  | |  |  | |  | |  | |  | |  | |  |
|  | | Paracetamol doses per week | | |  |  | |  | |  | | |  | |  | |  | |  | |  | |
|  | Con1 | | Con2 | Con3 | | | Con4 | | AIP1 | | AIP2 | AIP3 | | AIP4 | | AIP5 | | AIP6 | | AIP7 | | AIP8 |
| Ppt 1 | 1 | | 0 | 0 | | | 0 | | 0 | | 0 | * | | * | | 1 | | 0 | | 0 | | 1 |
| Ppt 2 | 0 | | 0 | 0 | | | 0 | | 0 | | 0 | 0 | | 0 | | 0 | | 0 | | 0 | | 0 |
| Ppt 3 | 0 | | 0 | 0 | | | 0 | | 0 | | 0 | 0 | | 0 | | 0 | | 0 | | 0 | | 0 |
| Ppt 4 | 0 | | 0 | 4 | | | 0 | | 2 | | 2 | 0 | | 0 | | 0 | | 0 | | 0 | | 0 |
| Ppt 5 | 0 | | 0 | 0 | | | 0 | | 0 | | 0 | 0 | | 0 | | 0 | | 0 | | 0 | | 0 |
| Ppt 6 | 3 | | 4 | 1 | | | 4 | | 0 | | 0 | 0 | | 2 | | 0 | | 1 | | 2 | | 0 |
| Ppt 7 | 7 | | 6 | 3 | | | 7 | | 3 | | 5 | 7 | | 7 | | 13 | | 12 | | 11 | | 12 |
| Ppt 10 | 0 | | 0 | 0 | | | 2 | | 1 | | 0 | 4 | | 1 | | 0 | | 6 | | 1 | | 7 |
| Ppt 11 | 6 | | 4 | 6 | | | 6 | | 3 | | 2 | 1 | | 0 | | 3 | | 2 | | 1 | | 0 |
| Mean | 1.89 | | 1.56 | 1.56 | | | 2.11 | | 1 | | 1 | 1.5 | | 1.25 | | 1.89 | | 2.33 | | 1.67 | | 2.22 |
| Mean usual diet |  | |  |  | | | 1.78 | |  | |  |  | |  | |  | |  | |  | |  |

**Notes:**

***Participant 1** had a back injury week 3-4 AIP and pain meds including added codeine were significant, and not related to RA so were not entered; Week 3 and 4 as follows:

- Week 3: Brufen SRx4, Nurofen 400x4, paracetamol x8, Norflex x3, codeine x2
- Week 4: paracetamol x 20, BrufSrx5, norflex x6, codeine x2, brufenx2

**Table S3: Symptoms associated with diet, summary**

Participants recorded weekly any symptoms they associated with their diet, both positive and negative. **Table S3** provides a summary of positive and negative effects attributed to diet, as documented in weekly participant feedback.

**Table S3:** Summary of positive and negative diet effects associated with diet during the study

| Participant | Baseline, control 4 weeks | AIP weeks 1-4 | AIP weeks 5-8 |
| --- | --- | --- | --- |
| 1  Negative | Intermittent bloating, indigestion. | Headaches 2 weeks, loose stools bloating weeks 1-3 | Minor IBS symptoms weeks 5-7 |
| Positive |  |  | Clearer skin, less rosacea. Weeks 6-8, less inflammation, good energy levels |
| 2  Negative | Acidic, occasional, IBS | Headaches, stomach pain, IBS, bad hot flushes week 1. Sugar cravings, slight headaches, week 2 | Stomach upset and headache after Covid vax week 5, otherwise no gut symptoms or headaches |
| Positive |  | Week 2-4, hot flushes decreased by 80%. More energy, sleeping better. Less congested “I can breathe!” IBS all but gone, settled in first week. Eyes not so gritty and itchy. Energy level higher. Headaches an odd event. | Virtually no hot flushes. A lot more energy. Feeling 200000% better. ITB not a problem (week8). Sleeping better. Walking 2-3 K daily without any problem |
| 3  Negative | None | None | Persistent diarrhoea, gurgling stomach weeks 6-8 |
| Positive |  |  | Better skin week 4-7 |
| 4  Negative | Intermittent heartburn, diarrhoea, constipation. | Headaches, skin breakouts, acne, increased inflammation week 1-2. breakouts week 3-4 | Breakouts week 6. Acne, hives week 7. |
| Positive |  | No digestive symptoms | More energy. No digestive symptoms |
| 5  Negative | None | Headaches, week 1, digestive system changes week 1,2,4. | Dry skin noticed week 5-8, no digestive symptoms |
| Positive |  | Week 3; reduced arthritic pain | Generally feel healthier |
| 6  Negative | None | Constipation week 1-3, low energy week 1 and 3 | Intermittent constipation and rosacea weeks 4-8 |
| Positive |  | Week 1-2 no headaches | More energy, almost no headaches |
| 7  Negative | Bloating, flatulence, alternating constipation and diarrhoea | Headaches first 2 days. Flatulence, bloating, weeks 1-3 | Gut changes, fatigue after dried fruit binge week 5, otherwise no digestive symptoms |
| Positive |  | Bowel movements improved week 2. Not noticing pain when sleeping. Skin glowing week 3-4 | Best sleep in a year. Less pain and swelling in joints, pain changed, arthritic damage not inflammation pain. Completely different person |
| 10  Negative | None | Loose stools intermittent week 1-2. Losing a little weight, unintentional | Loss of appetite, more unwanted weight loss, lethargic weeks 6-8 |
| Positive |  |  | Digestive system good, no bloating tummy like I used to sometimes have, week 7-8 |
| 11  Negative | Reflux and cough, persistent | Reflux, cough, lightheaded, headaches, ‘more gassy’ week 1. Irregular bowel motions, constipation week 2-4 | Intermittent irregular bowel motions, intermittent headache week 4-8 |
| Positive |  | Less bloated, week 1-4. Week 3-4 less inflammation in hands. | Less inflammation, more energy, lighter, easier to walk, run & bike |

**Figures S1 and S2: VAS results for Fatigue and Sleep, extracted from RAID**

**Fatigue**

Fatigue VAS scores were extracted from the RAID PRO questionnaire, shown in Figure S2. Mean fatigue scores decreased from 4.44 (range: 0–10) at baseline to 1.11 (range: 0–4) at week 12 (Figure 5). Eight of nine participants reported a final score of 0 or 1. Participant 4, who also had a diagnosis of fibromyalgia, reported a substantial fatigue reduction from 10 to 1. One participant reported fatigue that increased during the study.

**Figure S1** Weekly individual fatigue scores over 12 weeks


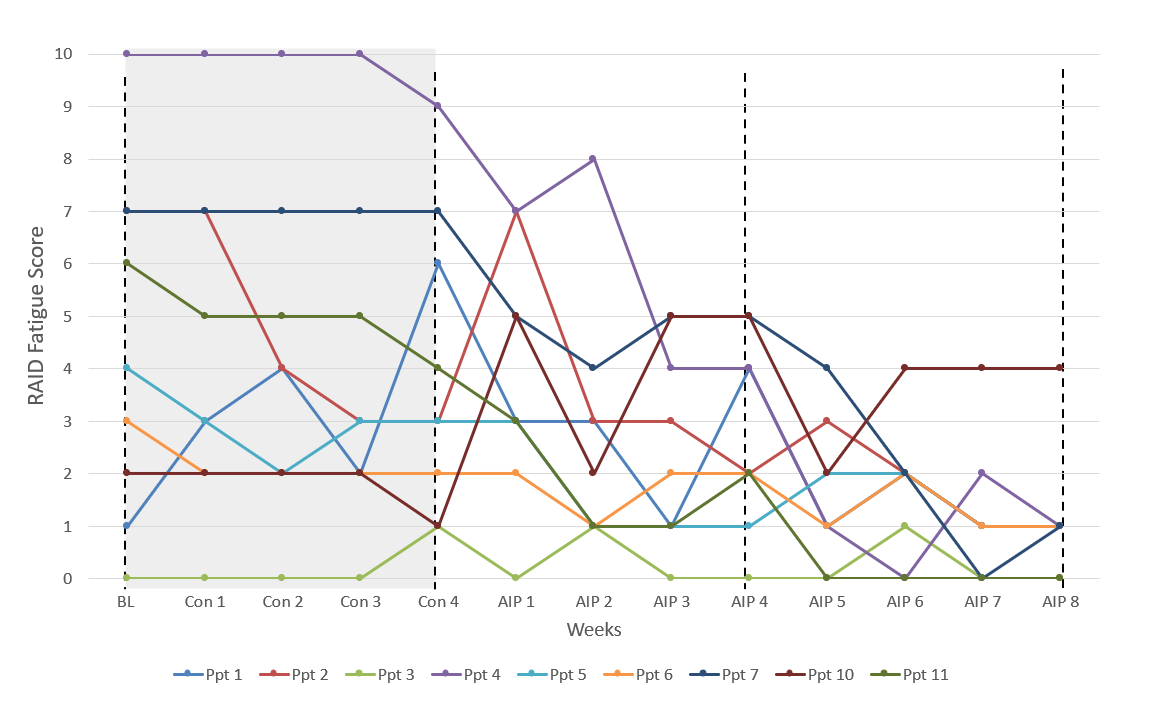


**Sleep**

Weekly VAS sleep scores were also extracted from RAID data, as shown in Figure S3. The mean sleep score decreased from 3.77 (range: 0–10) at baseline to 1.22 (range: 0–2) by week 12.

**Figure S2:**  Individual weekly sleep scores over 12 weeks


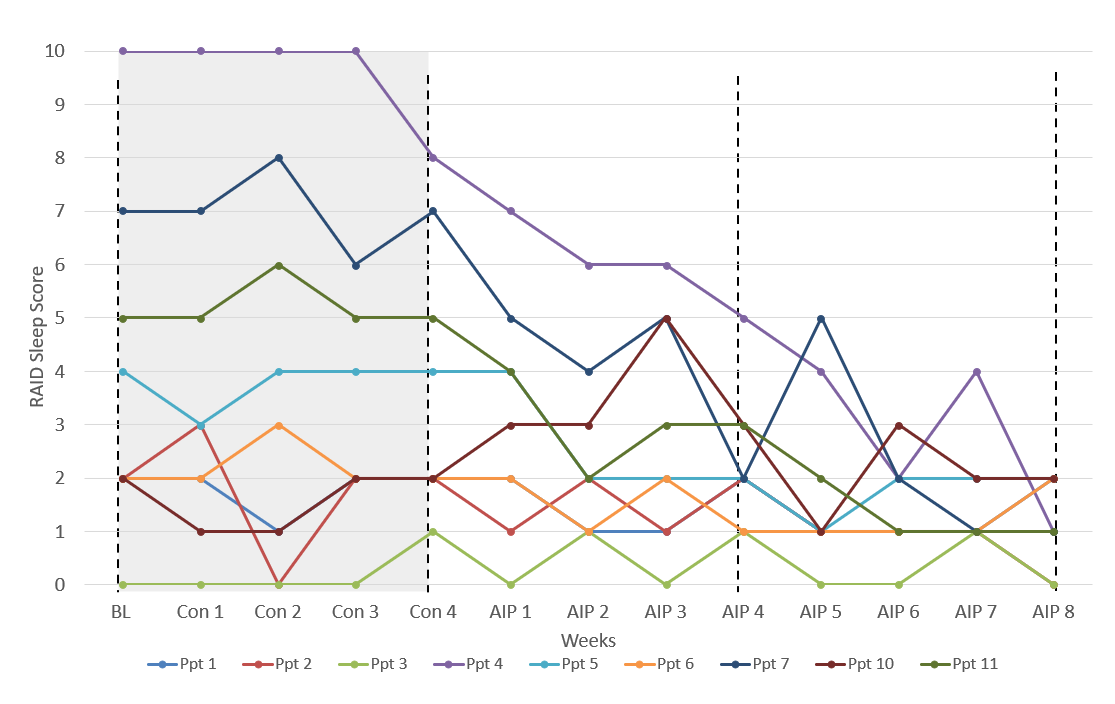

Supplement: Supplementary file 1 — Supporting Information S1 [file MSC-24-e70214-s001.docx]
